# 

**Published:** 1825-10

**Authors:** 


### MONTHLY CATALOGUE OF MEDICAL BOOKS.

*** *It having been hinted to us that gentlemen, sending copies of their Works, prefer having their titles given at length, it is our intention, in future, to comply with this suggestion: but it is to be observed, that no books can be entered on this List except those sent to us for the purpose;—finding, in the lists hitherto transmitted, that the names of works have frequently been given as published, which have not appeared for weeks, or even months, after.*

Medico-Chirurgical Transactions. Vol. XIII. Part I.—London, 1825.

Observations on Gout, Critical and Pathological; or, an Analytical Survey of the Views at present entertained of the Nature of that Disorder; with Practical Remarks on the Injurious Effects of Colchicum, and on certain Modes of Diet. By A. Rennie, Surgeon, &c.—London, 1825.

Observations on the Cholera Morbus of India: a Letter addressed to the Honourable the Court of Directors of the East-India Company. By Whitelaw Ainslie, M.D. M.R.A.S. late of the Medical Staff of Southern India.—London, 1825.

An Inquiry into the Seat and Nature of Fever; as deducible from the Phenomena, Causes, and Consequences of the Disease, the Effects of Remedies, and the Appearances on Dissection. By Henry Clutterbuck, M.D. Member of the Royal College of Physicians of London; senior Physician to the General Dispensary; Lecturer on the Theory and Practice of Medicine, &c. &c. Second Edition.—London, 1825.

A List of Drugs and Chemicals, including the new Medicines; Horse and Cattle Medicines, Perfumery, and other Articles generally sold by Chemists and Druggists; arranged alphabetically under their English Names, with the Latin Synonymes in general Use, and also the altered Names in the new Pharmacopœia; to which are added the Doses. Intended as a Price-Book. By a Chemist and Druggist, Author of the Apothecaries' Chart.—London, 1825.

Medical Researches on the Effects of Iodine, in Bronchocele, Paralysis, Chorea, Scrofula, Fistula Lachrymalis, Deafness, Dysphagia, White Swelling, and Distortions of the Spine. By Alexander Manson, M.D. Physician to the General Hospital, and St. Mary's Hospital and Dispensary, Nottingham.—London, 1825.

A Short Inquiry into the Capillary Circulation of the Blood; with a comparative View of the more intimate Nature of Inflammation: and an Introductory Essay. By James Black, M.D. S.R.N. and Member of the Royal College of Physicians of London.—London, 1825.

### NOTICE TO CORRESPONDENTS.

*The Papers of* Mr. Dix *(through* Dr. J. Johnson), Dr. Whymper, *and* Mr. Oswald, *have been received. We hope to hear again soon from* Mr. O.

Dr. Stoker *will find that we acknowledged the receipt of his Communication in our last.*

